# Supplementary material for: Optimization of Photogenerated Charge Carrier Lifetimes in ALD Grown TiO2 for Photonic Applications
Source: Nanomaterials (Basel). 2020 Aug 10;10(8):1567. doi: 10.3390/nano10081567 (PMC7466613; doi:10.3390/nano10081567)
Supplement: Supplementary file 1 [file nanomaterials-10-01567-s001.pdf]

Optimization of Photogenerated Charge Carrier Lifetimes in ALD Grown TiO_2 for Photonic Applications

Ramsha Khan [1,*], Harri Ali-Löytty [2], Jesse Saari [2], Mika Valden [2], Antti Tukiainen [3], Kimmo Lahtonen [3] and Nikolai V. Tkachenko [1,*]

1. Photonic Compounds and Nanomaterials group, Faculty of Engineering and Natural Sciences, Tampere University, P.O. Box 692, 33014, Tampere University, Tampere, Finland
2. Surface Science Group, Faculty of Engineering and Natural Sciences, Tampere University, P.O. Box 692, 33014, Tampere University, Tampere, Finland; harri.ali-loytty@tuni.fi (H.A.-L.); jesse.saari@tuni.fi (J.S.); mika.valden@tuni.fi (M.V.T.)
3. Faculty of Engineering and Natural Sciences, Tampere University, P.O. Box 692, 33014, Tampere University, Tampere, Finland; antti.tukiainen@tuni.fi (A.T.); kimmo.lahtonen@tuni.fi (K.L.)

* Correspondence: ramsha.khan@tuni.fi (R.K.); nikolai.tkachenko@tuni.fi (N.V.T.); Tel.: +358-407-484-160 (N.V.T.)

Supporting information

Results

The UV-Vis spectroscopy analysis for the sample series deposited at 100 ℃ and heat treated at different temperatures is shown in Figure S1.

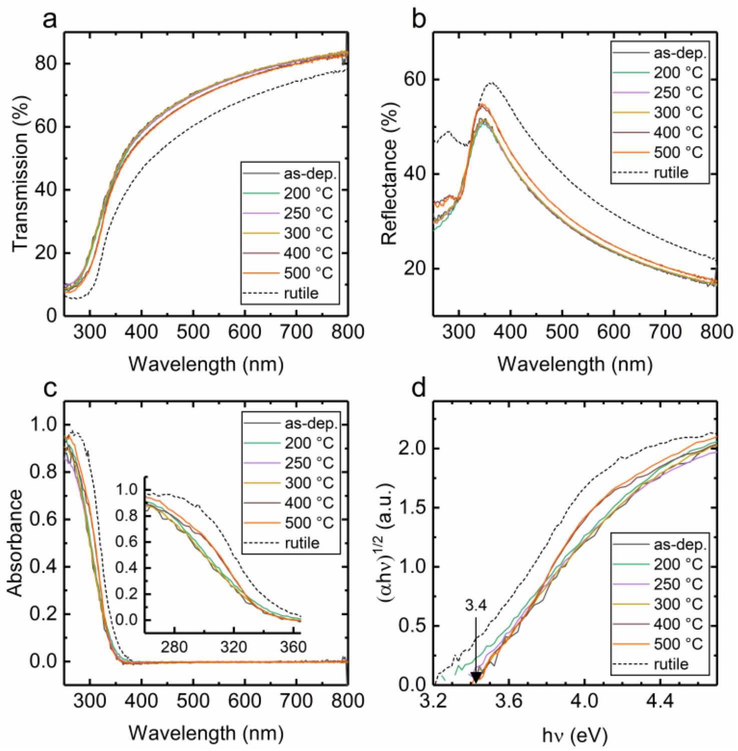

Figure S1: a) Transmittance spectra, (b) reflectance spectra, (c) absorbance spectra and (d) Tauc plots of the ALD TiO_2 thin films grown at 100 ℃ and heat treated at different temperatures indicated in the plots.

TAS data were acquired in transmittance and reflectance modes in the NIR region. The results are shown for samples deposited at 150 ℃ and heat treated at 200 ℃ and 500 ℃, i.e. before and after the onset of anatase phase, respectively, as shown in Figure S2. When sample is heat treated at 200 ℃, both transmittance and reflectance show almost same intensity, Figure S2(a,b). When heat treatment temperature is increased to 500 ℃, the reflected signal is much stronger than the transmitted one, Figure S2(c,d). This effect can be dedicated to difference in crystallinity and different relative chance of refractive index and absorption coefficient after excitation, respectively.

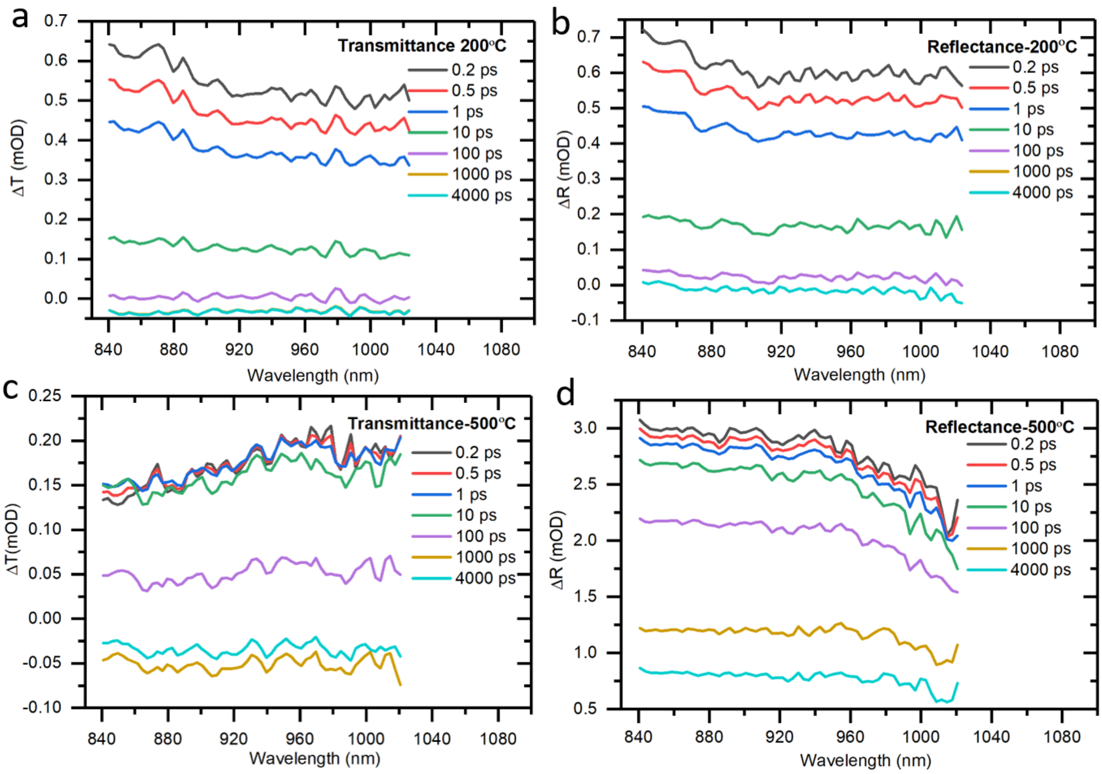

Figure S2: TA spectra in transmittance and reflectance modes for (a,b) 200 ℃ and (c,d) 500 ℃ heat-treated samples, respectively

Carrier relaxation time:

To compare the carrier relaxation time the TA data were fitted by a sum of exponent and stretched-exponent. The former was needed to account for the fast carrier thermalization which takes place in the subpicosecond time domain and almost invisible in Figure 3, which presents TA in longer time domain (from 0.1 ps to 5 ns). Thus the carrier recombination is presented by single stretched-exponential

$$A(t) = a_0 exp\left[-\left(\frac{t}{\tau}\right)^{\beta}\right]$$

where τ is the time constant and β is the stretching parameter. Since the stretching parameter was somewhat different for different samples the average lifetime was calculated as by Ref [1], which refers to Ref [2].

$$\tau_{av} = \frac{\tau}{\beta}\Gamma\left(\frac{1}{\beta}\right)$$

where $\Gamma(\quad)$ is the Gamma-function.

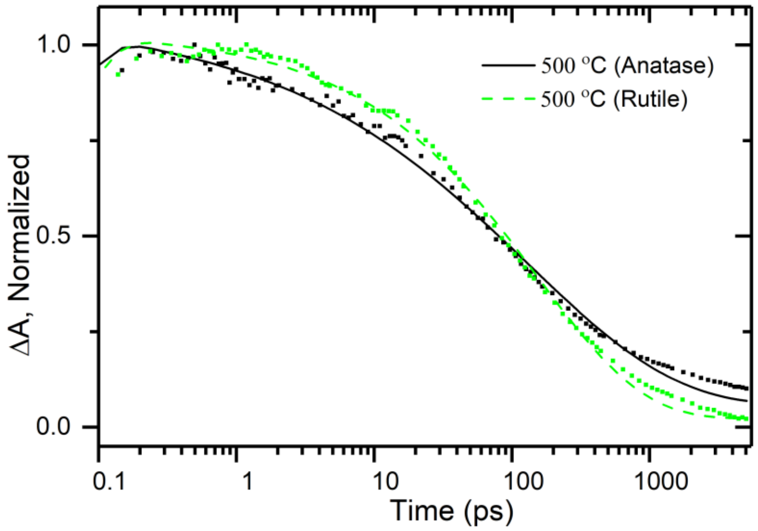

Figure S3: Comparison of lifetime of 150 ℃ deposited, 500 ℃ heat-treated sample (anatase) and 200 ℃ deposited, 500 ℃ heat-treated sample (rutile)

References:

1. Schlegel, G.; Bohnenberger, J.; Potapova, I.; Mews, A. Fluorescence Decay Time of Single Semiconductor Nanocrystals. *Phys. Rev. Lett.* **2002**, *88*, 4, doi:10.1103/PhysRevLett.88.137401.
2. Lindsey, C.P.; Patterson, G.D. Detailed comparison of the Williams-Watts and Cole-Davidson functions. *J. Chem. Phys.* **1980**, *73*, 3348–3357, doi:10.1063/1.440530.
